# Rhythm outcomes after aortic valve surgery: Treatment and evolution of new‐onset atrial fibrillation

**DOI:** 10.1002/clc.23703

**Published:** 2021-08-14

**Authors:** Bitao Xiang, Wenrui Ma, Shixin Yan, Jinmiao Chen, Jun Li, Chunsheng Wang

**Affiliations:** ^1^ Department of Cardiac Surgery, Shanghai Institute of Cardiovascular Diseases, Zhongshan Hospital Fudan University Shanghai China

**Keywords:** aortic valve, atrial fibrillation, surgery

## Abstract

**Background:**

The impact of new‐onset atrial fibrillation (AF) after aortic valve (AV) surgery on mid‐ and long‐term outcomes is under debate. Here, we sought to follow up heart rhythms after AV surgery, and to evaluate the mid‐term prognosis and effectiveness of treatment for patients with new‐onset AF.

**Methods:**

This single‐center cohort study included 978 consecutive patients (median age, 59 years; male, 68.5%) who underwent surgical AV procedures between 2017 and 2018. All patients with postoperative new‐onset AF were treated with Class III antiarrhythmic drugs with or without electrical cardioversion (rhythm control). Status of survival, stroke, and rhythm outcomes were collected and compared between patients with and without new‐onset AF.

**Results:**

New‐onset AF was detected in 256 (26.2%) patients. For them, postoperative survival was comparable with those without new‐onset AF (1‐year: 96.1% vs. 99.3%; adjusted *P* = .30), but rate of stroke was significantly higher (1‐year: 4.0% vs. 2.2%; adjusted *P* = .020). With rhythm control management, the 3‐month and 1‐year rates of paroxysmal or persistent AF between patients with and without new‐onset AF were 5.1% versus 1.3% and 7.5% versus 2.1%, respectively (both *P* < .001). Multivariate models showed that advanced age, impaired ejection fraction, new‐onset AF and discontinuation of beta‐blockers were predictors of AF at 1 year.

**Conclusions:**

In most cases, new‐onset AF after AV surgery could be effectively converted and suppressed by rhythm control therapy. Nevertheless, new‐onset AF predisposed patients to higher risks of stroke and AF within 1 year, for whom prophylactic procedures and continuous beta‐blockers could be beneficial.

## 1 INTRODUCTION

New‐onset atrial fibrillation (AF) after cardiac surgery is a common complication that is associated with major adverse events.^1^, ^2^ It has been well reported that new‐onset AF after aortic valve (AV) procedures elevates in‐hospital mortality, while its sole effect on long‐term endpoints remain controversial.^3^, ^4^, ^5^ It should be noted that most of the studies attempted to relate new‐onset AF to late events directly, without detailed heart rhythm follow‐ups to support the causal relationship.

Regarding the optimal management of postoperative new‐onset AF, current guidelines and recent trials recommend both rate control and rhythm control therapies.^1^, ^6^ At our institution, rhythm control is the preferred choice, and conversion is promptly performed for new‐onset AF. However, there is a lack of clinical and rhythm data to validate the mid‐term effectiveness of such strategy. Furthermore, in addition to the well‐known predictors of postoperative new‐onset AF (e.g., age and left atrial size), risk factors that may predispose patients to long‐term AF after AV surgery are also clinically relevant.

Hence, in this study, we aimed to (1) follow up heart rhythm after AV surgery; (2) compare clinical and rhythm outcomes between those with rhythm controlled new‐onset AF and those who remained in sinus rhythm throughout postoperative hospitalization; and (3) identify predictors of paroxysmal or persistent AF at 1 year.

## 2 PATIENTS AND METHODS

### 2.1 Study population

**FIGURE 1:**
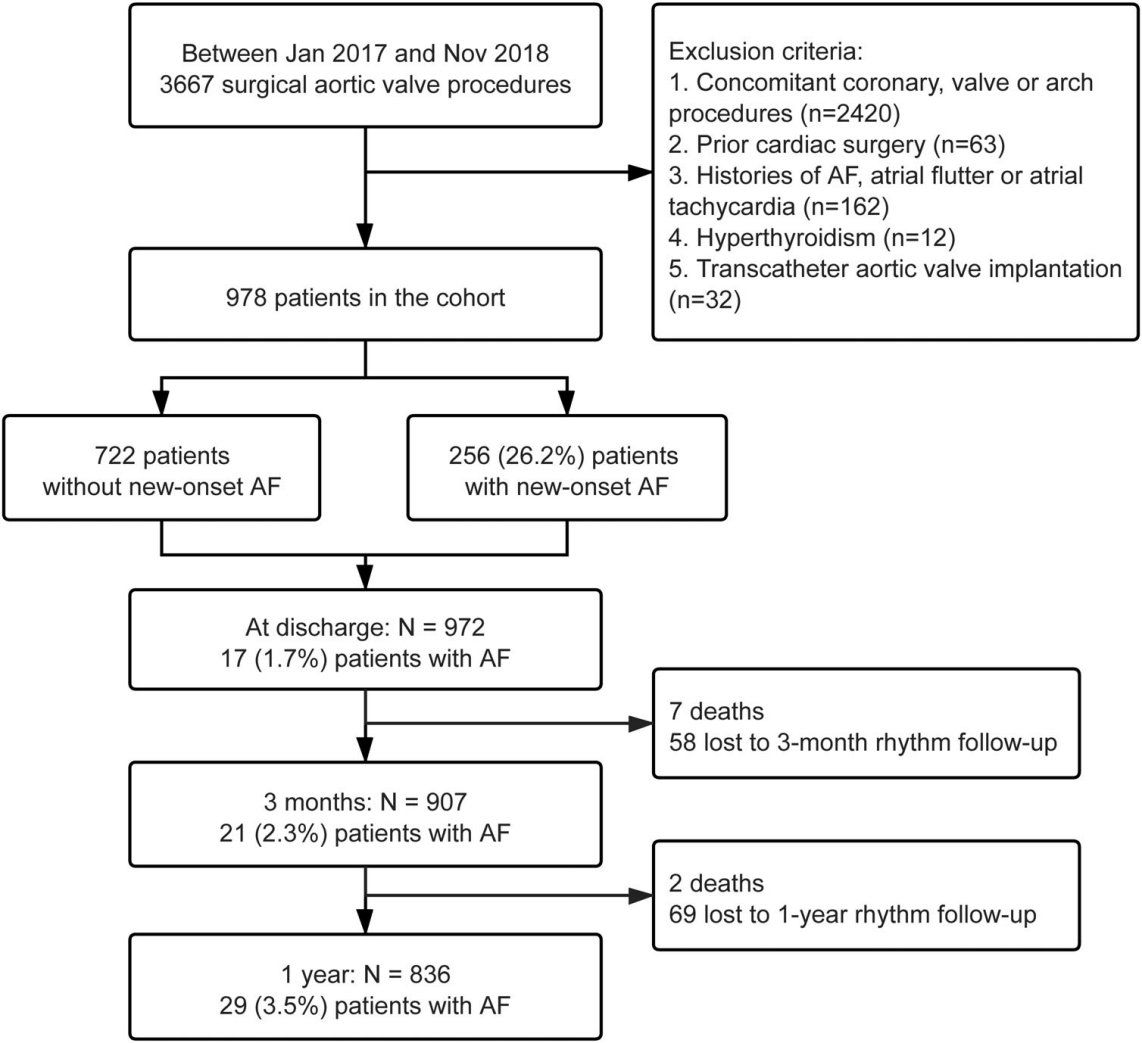
Flowchart presenting the inclusion criteria and rhythm outcomes. AF, atrial fibrillation

This study was approved by the Ethics Committee of Patients of Zhongshan Hospital. The requirement for informed consent was waived because of the retrospective nature of the study (approval number: B2021‐534R). Between January 2017 and November 2018, data of 3667 consecutive patients undergoing AV surgery at our Department of Cardiac Surgery (Zhongshan Hospital Fudan University, Shanghai, China) were reviewed. We excluded patients with histories of AF, atrial flutter or atrial tachycardia, hyperthyroidism, and those who underwent cardiac reoperations. Concomitant procedures were limited to root reconstruction and ascending aortic repair. Patients undergoing transapical transcatheter AV implantation were also excluded. After screening, 978 patients were selected as the study cohort (Figure 1).

### 2.2 Operative procedures

All patients underwent AV replacement or repair, with concomitant procedures limited to root reconstruction and ascending aortic repair. Patients with concomitant coronary artery, other valve or aortic arch procedures were excluded. The details of surgical procedures were reported in previous studies.^7^, ^8^ Invasive approaches included sternotomy (*n* = 871, 89.1%), supra‐sternotomy (*n* = 79, 8.1%) and minimally invasive right thoracotomy (*n* = 28, 2.9%). The left ventricle was routinely vented via right superior pulmonary vein. Bioprostheses were implanted in 360 (36.8%) patients. No patient received prophylactic pulmonary vein isolation or ligation of left atrial appendage.

### 2.3 New‐onset AF: Rhythm control therapy

At our institution, we did not prophylactically use Class III antiarrhythmic drugs. Instead, preoperative beta‐blockers were routinely administered in all patients planned for AV surgery, unless contraindicated. The heart rhythm of each patient was monitored continuously from postoperative day 0 until discharge using telemetry. New‐onset AF was defined as postoperative AF that lasted at least for 30 s or recurred during hospitalization.^9^ Whenever a period of AF was noted by the nursing staff, the physician on‐call would be informed and respond accordingly. Data of the rhythm control treatment, including intravenous and/or oral use of Class III antiarrhythmic drugs with and without beta‐blockers and electrical cardioversion, were collected by interrogating records of daily rounds and order lists.

Warfarin therapy was initiated on postoperative day 0 in all patients, and doses were titrated to achieve an international normalized ratio of 2.0–3.0. Patients with new‐onset AF that was not successfully converted were discharged on continuous oral amiodarone or sotalol for 3 months. Antiarrhythmic drugs were discontinued in patients with completed 3‐month regimen and in those who developed drug‐related side effects, including dizziness, blurred vision, cough, dyspnea, symptomatic bradycardia, electrocardiogram‐confirmed long‐QT syndrome or ventricular tachycardia.

Heart rhythm follow‐ups included electrocardiogram, 24‐h Holter monitoring and pacemaker interrogation, which were performed at the discretion of the referring cardiologist and the operating surgeon. Specifically, electrocardiogram and pacemaker interrogation were repeated at 1, 3, 6, and 12 months after surgery, and yearly thereafter. Patients who complained of paroxysmal/continuous palpitations received 24‐h Holter monitoring. Additional Holter monitoring was performed if previous reports did not reveal AF while the symptoms persisted.

For patients who underwent bioprosthetic AV replacement or valve repair, anticoagulation was discontinued if sinus rhythm was stable at 3 months. If AF was still detectable at 3 months, electrical cardioversion or transcatheter ablation was recommended. Rate control and anticoagulation therapies were used in patients with persistent AF who had undergone at least 1 electrical cardioversion or transcatheter ablation, and in those who refused those procedures.

### 2.4 Follow‐up

The primary outcomes of this study were all‐cause mortality and stroke. The secondary outcomes were heart rhythm statuses at 3 months and at 1 year after surgery. Data of patient status and therapy after discharge were prospectively collected via telephone calls and the outpatient clinic database. Collection of follow‐up data was performed between November 1, 2019 and January 15, 2020. Clinical follow‐up was 93.9% (918/978) complete at a median of 19 months (interquartile range, 14–25 months). The baseline and perioperative data were compared between patients with and without follow‐up data (Table S1).

Heart rhythm data were available in 93.8% (*n* = 907) survivors at 3 months, and in 92.1% (*n* = 836) survivors at 1 year, including a total of 2588 electrocardiograms, 65 Holter monitoring reports and 30 pacemaker interrogations during follow‐up (median time [interquartile range], 15 [12–24] months). All patients with rhythm follow‐ups had at least two electrocardiograms. In this study, data of survival and rhythm outcomes were collected by two independent investigators (B.X. and S.Y.) who were blinded to the baseline and perioperative data.

### 2.5 Statistical analysis

Continuous variables were presented as mean ± SD and compared using the Student *t*‐test or Mann–Whitney *U*‐test according to the Shapiro–Wilk normality test. Categorical variables were described as numbers and percentages and analyzed using the chi‐square test or Fisher's exact test, as appropriate. The baseline and perioperative data were complete in all patients.

Multivariate logistic regression models were used to identify predictors of new‐onset AF with the forward stepwise selection method (*P*_entry_ = .10, *P*_stay_ = .05). The baseline and operative covariates included in the models were age, gender, comorbidities, New York Heart Association functional class III–IV, baseline left atrial dimension, left ventricular ejection fraction, AV pathologies, operating procedures (surgery vs. intervention, valve repair vs. replacement and concomitant procedures), type and size of prosthesis, in‐hospital morbidities, transfusion, hypokalemia, and perioperative inotropic agents. Covariates with *P* < .10 in univariate models were included in the multivariate model. The Hosmer–Lemeshow goodness‐of‐fit test and calculation of c‐statistic were performed for the final model (model 1).

Kaplan–Meier curves were used to describe freedom from death and stroke. To compare survival between patients with and without new‐onset AF, the inverse probability weighting method was used to adjust multiple covariates, including age, gender, coronary artery disease, diabetes, hypertension, chronic lung disease, and cerebrovascular disease, New York Heart Association functional class III–IV, left atrial dimension, left ventricular ejection fraction, surgical approaches and procedures. Competing risks of mortality and stroke were analyzed using the Fine–Gray method.

Multivariate logistic regression models were also established to investigate predictors of AF at 1 year. Apart from the covariates listed above, non‐antiarrhythmic medications that were administered after 3 months, including loop diuretics, spironolactone, beta‐blockers, angiotensin‐converting enzyme inhibitor/angiotensin receptor blockers, digoxin and statins, were also included in the models (model 2).

A two‐tailed *P* value <.05 was considered statistically significant. All statistical analyses were performed using R v3.3.3 (Package “IPWsurvival”, R Development Core Team, Vienna, Austria) and STATA 15 (StataCorp LP, TX, USA).

## 3 RESULTS

### 3.1 Patient demographics

The baseline, operative and postoperative characteristics were listed in Tables 1 and 2. The overall mean age was 59 years (range, 18–90 years) with 68.5% male patients. The overall in‐hospital rates of mortality and stroke were 0.6% (*n* = 6) and 1.4% (*n* = 14), respectively. Postoperative new‐onset AF occurred in 256 (26.2%) patients, which was associated with higher risks of in‐hospital mortality (1.6% vs. 0.3%, *P* = .044). Rate of in‐hospital stroke was comparable (2.3% vs. 1.1%, *P* = .22).

**TABLE 1:** Baseline and perioperative characteristics of the entire cohort and comparisons between patients with and without new‐onset AF

Demographics	All (*N* = 978)	No AF (*n* = 722)	AF (*n* = 256)	*P* value
Male sex	670 (68.5)	486 (67.3)	184 (71.9)	.18
Age	59 (48–66)	56 (46–64)	64 (55–69)	<.001
Body mass index (kg/m^2^)	23.7 (21.8–25.0)	23.4 (21.0–24.2)	24.3 (22.5–26.0)	.11
Diabetes	69 (7.1)	41 (5.7)	28 (10.9)	.005
Hypertension	415 (42.4)	283 (39.2)	132 (51.6)	<.001
Coronary artery disease	75 (7.7)	47 (6.5)	28 (10.9)	.022
Chronic lung disease	25 (2.6)	14 (1.9)	11 (4.3)	.040
Cerebrovascular disease	45 (4.6)	33 (4.6)	12 (4.7)	.94
Chronic kidney disease	23 (1.8)	14 (1.9)	4 (1.6)	>.99^a^
Peripheral artery disease	16 (1.6)	8 (1.1)	8 (3.1)	.042^a^
Connective tissue disorder	17 (1.7)	15 (2.1)	2 (0.8)	.26^a^
Autoimmune disease	23 (2.4)	18 (2.5)	5 (2.0)	.62
New York Heart Association functional class III–IV	734 (75.1)	527 (73.0)	207 (80.9)	.012
Heart rate (beats per minute)	69 (63–77)	69 (62–77)	69 (63–79)	.39
Left atrial dimension (mm)	40 (37–44)	40 (36–44)	42.8 ± 5.7	<.001
Left ventricular ejection fraction (%)	62 (56–66)	62 (57–66)	60 (51–65)	<.001
Ejection fraction <50%	121 (12.4)	69 (9.6)	52 (20.3)	<.001
Bicuspid AV	428 (43.8)	329 (45.6)	99 (38.7)	.056
Aortic stenosis	181 (18.5)	129 (17.9)	52 (20.3)	.39
Aortic regurgitation	539 (55.1)	406 (56.2)	133 (52.0)	.24
Aortic stenosis + regurgitation	258 (26.4)	187 (25.9)	71 (27.7)	.57
Mitral regurgitation >mild	22 (2.2)	15 (2.1)	7 (2.7)	.54
Tricuspid regurgitation >mild	15 (1.5)	9 (1.3)	6 (2.3)	.24^a^
Ascending aortic diameter > 40 mm	521 (53.3)	374 (51.8)	147 (57.4)	.12
EuroSCORE II (%)	1.34 ± 0.26	1.28 ± 0.20	1.51 ± 0.33	<.001

*Note*: Continuous variables are presented as medians (interquartile ranges) or means ± SD, according to the normality test. Categorical variables are presented as numbers (percentages).

Abbreviations: AF, atrial fibrillation; AV, aortic valve.

^a^ Fisher's exact test.

**TABLE 2:** Operative and postoperative characteristics of the entire cohort and comparisons between patients with and without new‐onset AF

Demographics	All (*N* = 978)	No AF (*n* = 722)	AF (*n* = 256)	*P* value
Cardiopulmonary bypass time (min)	91 (70–111)	89 (68–108)	101.5 ± 31.7	.070
Aortic cross‐clamp time (min)	57 (45–77)	56 (45–75)	58 (47–82)	.23
Approaches				
Sternal	871 (89.1)	629 (87.1)	242 (94.5)	.001
Supra‐sternal	79 (8.1)	67 (9.3)	12 (4.6)	.021
Right‐thoracic	28 (2.9)	26 (3.6)	2 (0.8)	.020
Bioprosthesis	360 (36.8)	222 (30.8)	138 (53.9)	<.001
Prosthetic size	23 (21–25)	23 (21–25)	23 (21–25)	.28
AV repair	62 (6.3)	53 (7.3)	9 (3.5)	.031
Effective orifice area index <0.85 cm^2^/m^2^	111 (11.3)	77 (10.7)	34 (13.3)	.26
Ascending aortic/root replacement	347 (35.5)	250 (34.6)	97 (37.9)	.35
In‐hospital mortality	6 (0.6)	2 (0.3)	4 (1.5)	.044^a^
Length of stay in the intensive care unit				
Morbidities	51 (5.2)	34 (4.7)	17 (6.6)	.23
Low cardiac output syndrome	11 (1.1)	8 (1.1)	3 (1.2)	>.99^a^
Stroke	14 (1.4)	8 (1.1)	6 (2.3)	.22^a^
Dialysis	6 (0.6)	1 (0.1)	5 (1.9)	.006^a^
Ventilator support >96 h	17 (1.7)	9 (1.3)	8 (3.1)	.089^a^
Reoperation for bleeding	2 (0.2)	2 (0.3)	0 (0)	>.99^a^
Pacemaker implantation	17 (1.7)	14 (1.9)	2 (0.8)	.26^a^
Hypokalemia	185 (18.9)	112 (15.5)	73 (28.5)	<.001
Transfusion	233 (23.8)	155 (21.5)	78 (30.2)	.004
Red blood cell (IU)	0 (0–0)	0 (0–0)	0 (0–2)	.002
Plasma (ml)	0 (0–0)	0 (0–0)	0 (0–400)	.003
Perioperative medications				
Epinephrine/norepinephrine	876 (89.6)	643 (89.1)	233 (91.0)	.38
Phosphodiesterase inhibitor	922 (94.3)	682 (94.5)	240 (93.8)	.67
Dopamine/dobutamine	330 (33.7)	208 (28.8)	122 (47.7)	<.001
Levosimendan	49 (5.0)	31 (4.3)	18 (7.0)	.085

*Note*: Continuous variables are presented as medians (interquartile ranges) or means ± SD, according to the normality test. Categorical variables are presented as numbers (percentages).

Abbreviations: AF, atrial fibrillation; AV, aortic valve.

^a^ Fisher's exact test.

### 3.2 New‐onset AF and predictors

Treatments of new‐onset AF included Class III antiarrhythmic drugs (*n* = 256), beta‐blockers (*n* = 17) and electric cardioversion (*n* = 4) to restore sinus rhythm. As a result, 235 (93.3%) patients were successfully converted to sinus rhythm before discharge.

The final multivariate logistic model (model 1) showed that age per year (odds ratio [OR], 1.05; 95% confidence intervals [CI], 1.04–1.07; *P* < .001); baseline left atrial dimension per mm (OR, 1.06; 95% CI, 1.03–1.09; *P* < .001), left ventricular ejection fraction per % (OR, 0.98; 95% CI, 0.97–1.00; *P* = .045), hypokalemia (OR, 1.82; 95% CI, 1.26–2.62; *P* = .001) and perioperative use of dopamine/dobutamine (OR, 1.81; 95% CI, 1.32–2.48; *P* < .001) were significantly associated with occurrence of new‐onset AF (c‐statistic = 0.740; Hosmer–Lemeshow test, *P* = .47).

### 3.3 Primary outcomes

**FIGURE 2:**
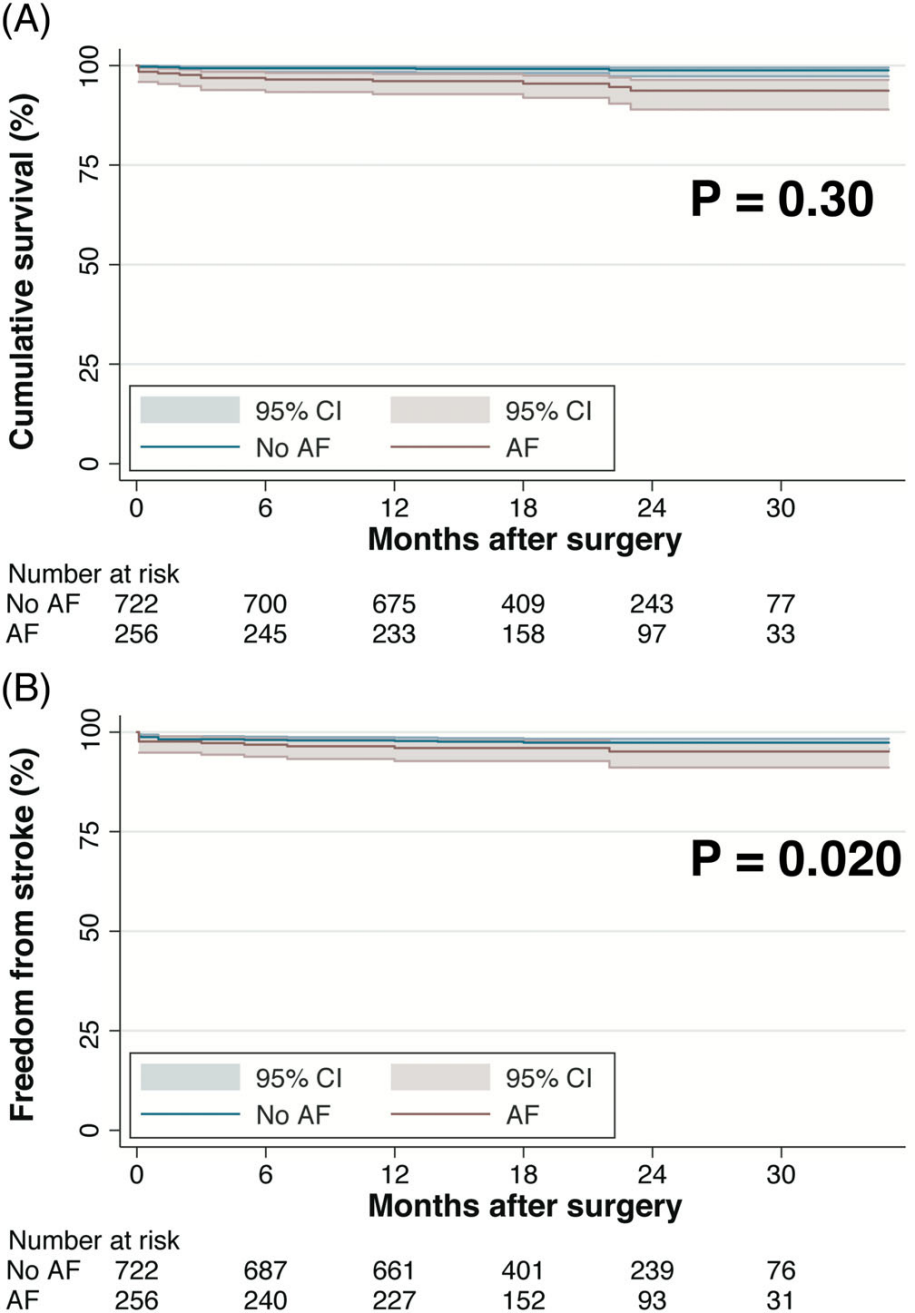
Kaplan–Meier curves presenting differences in survival (A) and freedom from stroke (B) between patients with and without new‐onset AF. AF, atrial fibrillation; CI, confidence interval

For the entire cohort, 14 (1.4%) deaths occurred after discharge, including 3 due to cerebrovascular events, 5 due to cardiac causes and 6 due to other causes. Stroke occurred in 15 (1.5%) patients after discharge. The 1‐year cumulative rates of mortality and stroke were 1.7 ± 0.4% and 2.7 ± 0.5%, respectively. Before adjustment, there was significant differences in mortality and stroke between patients with and without new‐onset AF (1‐year mortality, 3.9 ± 0.1% vs. 0.7 ± 0.03%; 1‐year rate of stroke, 4.0 ± 0.1% vs. 2.2 ± 0.1%; both *P* < .001; Figure 2). After adjustment, difference in stroke rate was still significant (subdistribution hazard ratio, 3.46; 95% CI, 1.22–9.84; *P* = .020), while mortality was comparable between patients with and without new‐onset AF (adjusted log‐rank, 1.13; *P* = .30).

### 3.4 Rhythm follow‐up and predictors of AF

Details of rhythm follow‐ups and medications during follow‐up were shown in Figure 1 and in Table 3, respectively. At 3 months, among 907 survivors with rhythm follow‐ups, 21 (2.3%) patients had paroxysmal or persistent AF, including 12 (5.1%) patients with postoperative new‐onset AF and 9 (1.3%) patients without (*P* < .001). Anticoagulation was continued in all those patients. Electric cardioversion and transcatheter ablation were performed in 5 and 2 patients with new‐onset AF, respectively. Class III antiarrhythmic drugs were administered in the 9 patients without postoperative new‐onset AF. Altogether, conversion was successful in 7 (33.3%) patients who were free from AF recurrence thereafter. At 1 year, among 836 survivors with rhythm follow‐ups, the rate of persistent AF was 3.5% (*n* = 29), including 16 (7.5%) patients with postoperative new‐onset AF and 13 (2.1%) patients without (*P* < .001). Patients were treated with long‐term anticoagulants after ineffective rhythm control therapy. Among patients undergoing bioprosthetic AV replacement or valve repair, prolonged anticoagulation was administered in 11 (2.7%) patients at 3 months and in 14 (3.8%) patients at 1 year. Adjusted models did not reveal significant difference in rates of mortality and stroke between patients receiving mechanical prostheses and those with bioprostheses or repaired AV (adjusted *P* = .67 and 0.20, respectively).

**TABLE 3:** Postoperative medications after AV surgery in patients with and without new‐onset AF

Drugs	3 months (*n* = 907)			1 year (*n* = 836)		
	No AF (*n* = 673)	AF (*n* = 234)	*P* value	No AF (*n* = 623)	AF (*n* = 213)	*P* value
Warfarin/aspirin therapy	78 (11.6)	44 (18.8)	.005	80 (12.8)	41 (19.2)	.022
Loop diuretics	653 (97.0)	225 (96.2)	.51	47 (7.5)	22 (10.3)	.20
Spironolactone	552 (82.0)	189 (80.8)	.67	80 (12.8)	34 (16.0)	.25
Beta‐blockers	513 (76.2)	158 (67.5)	.009	391 (62.8)	142 (66.7)	.31
Angiotensin‐converting enzyme inhibitor/angiotensin receptor blocker	54 (8.0)	19 (8.1)	.96	67 (10.8)	18 (8.5)	.37
Digoxin	5 (0.7)	3 (1.3)	.43^a^	9 (1.4)	4 (1.9)	.75^a^
Statins	35 (5.2)	21 (9.0)	.039	35 (5.6)	19 (8.9)	.091
Class III antiarrhythmic drugs	9 (1.3)	234 (100.0)	<.001	13 (2.1)	16 (7.5)	<.001

*Note*: Categorical variables are presented as numbers (percentages).

Abbreviations: AF, atrial fibrillation; AV, aortic valve.

^a^ Fisher's exact test.

Multivariate logistic regression models (model 2) showed that age (OR, 1.04; 95% CI, 1.01–1.08; *P* = .019), baseline left ventricular ejection fraction (OR, 0.97; 95% CI, 0.93–1.00; *P* = .027), new‐onset AF (OR, 2.56; 95% CI, 1.15–5.70; *P* = .022) and use of beta‐blockers after 3 months (OR, 0.45; 95% CI, 0.21–0.97; *P* = .041) were important predictors of persistent AF at 1 year (c‐statistic = 0.754; Hosmer–Lemeshow test, *P* = .80; Table 4).

**TABLE 4:** Univariate and multivariate logistic regression models to identify predictors of persistent AF at 1 year

Variables	Univariate models		Multivariate models	
	OR (95% CI)	*P* value	OR (95% CI)	*P* value
Age per year	1.05 (1.01–1.09)	.011	1.04 (1.01–1.08)	.019
Male sex	1.48 (0.62–3.50)	.38		
Coronary artery disease	0.94 (0.22–4.05)	.93		
Diabetes	1.41 (0.42–4.80)	.58		
Hypertension	1.08 (0.51–2.28)	.83		
Chronic lung disease	1.41 (0.18–10.85)	.74		
Cerebrovascular disease	2.40 (0.70–8.30)	.17		
New York Heart Association III–IV	2.16 (0.74–6.27)	.16		
Left atrial dimension per mm	1.05 (0.98–1.11)	.16		
Left ventricular ejection fraction per %	0.96 (0.93–0.99)	.010	0.97 (0.93–1.00)	.027
Mitral regurgitation > mild	2.02 (0.26–15.93)	.50		
Bicuspid AV	0.83 (0.39–1.77)	.62		
Sternotomy	3.43 (0.46–25.50)	.23		
Bioprosthesis	1.66 (0.79–3.48)	.18		
New‐onset AF	3.81 (1.80–8.06)	<.001	2.56 (1.15–5.70)	.022
Medication after 3 months				
Loop diuretics	‐	‐		
Spironolactone	1.09 (0.41–2.90)	.88		
Beta‐blockers	0.43 (0.20–0.90)	.026	0.45 (0.21–0.97)	.041
Angiotensin‐converting enzyme inhibitor/angiotensin receptor blocker	1.86 (0.63–5.50)	.26		
Digoxin	4.08 (0.49–34.31)	.20		
Statin	1.08 (0.25–4.65)	.92		

Abbreviations: AF, atrial fibrillation; AV, aortic valve; CI, confidence interval; OR, odds ratio.

## 4 DISCUSSION

The main findings of this study are twofold. First, by following up clinical and heart rhythm outcomes after AV surgery, we found that patients who developed new‐onset AF during hospitalization were at higher risks of postoperative stroke and AF at 1 year, compared with those without new‐onset AF. Second, rhythm control strategy was effective to restore sinus rhythm with a > 90% success rate at 1 year, which could be augmented by continuous use of beta‐blockers after 3 months.

It is accepted that new‐onset AF after cardiac surgery is a multifactorial complication associated with advanced age, comorbidities, cardiac dysfunction and neurohumoral disturbances.^10^, ^11^, ^12^ In addition, new‐onset AF is less common after transfemoral transcatheter AV implantation compared with standard sternotomy and non‐transfemoral approaches.^9^, ^13^, ^14^, ^15^ Consistently, in this study, age, left atrial dimension, left ventricular ejection fraction, hypokalemia and use of dopamine/dobutamine were identified as independent predictors of new‐onset AF. Apart from better control of electrolyte balance and avoidance of dopamine/dobutamine, prophylactic treatments, namely closure of left atrial appendage, pulmonary vein isolation or preoperative use of amiodarone, might be useful to reduce occurrence of new‐onset AF for patients with advanced age, left atrial enlargement and poor left ventricular systolic function.^16^

Over the past decade, characteristics and treatment of new‐onset AF after surgical and transcathether AV replacement have become a topic of interest. Some studies reported the adverse impact of new‐onset AF on early outcomes, including increased length of stay, risk of stroke, and in‐hospital mortality.^1^, ^2^, ^3^, ^4^, ^10^ In contrast, there were data showing that long‐term survival was not significantly impaired by the presence of new‐onset AF.^5^, ^17^ In the current study, after adjusting baseline and operative confounders, we found that new‐onset AF was not identified as an independent risk of mid‐term mortality. Nevertheless, despite being amenable in most cases, new‐onset AF was associated with remarkably elevated risks of postoperative stroke, persistent AF and prolonged anticoagulation within 1 year. Those results emphasized the importance of identifying and pretreating patients with high risk of persistent new‐onset AF.

Regarding the natural course of new‐onset AF after AV surgery, many cardiac surgeons believe that new‐onset AF after AV surgery is mostly transient and rarely develops into long‐term AF. Recently, Axtell et al introduced the term “new‐onset prolonged AF” to describe new‐onset AF that occurred within 30 days and persisted for at least 1 month after AV surgery.^16^ They reported a rate of new‐onset prolonged AF as high as 24%, highlighting the necessity to treat such complication. However, their strategy was not detailed in the article. Our rhythm follow‐up data were similar to those of a multicenter randomized trial conducted by Gillinov et al, who reported that 97.9% patients receiving rhythm‐control therapy were free from AF recurrence at 60 days after surgery (in our series, 94.9% at 3 months and 92.5% at 1 year), by which we conclude that rhythm control therapy is effective to convert and suppress new‐onset AF in most patients within 1 year. Regarding the safety of rhythm control therapy, the trial showed that 23.8% of the patients did not complete the full course of amiodarone due to drug‐related toxic effects.^5^ In our study, the rate of nonadherence was trivial because amiodarone was replaced by sotalol whenever drug‐related symptoms occurred, and duration of antiarrhythmic therapy (amiodarone, 200 mg q.d.; sotalol, 80 mg b.i.d.) was limited to 3 months.^18^

To investigate risk factors of AF at 1 year, we added medications prescribed within 3 months and after 3 months after surgery to the models. Our data demonstrated that continuous use of beta‐blockers after 3 months reduced the frequency of AF at 1 year. Given this, beta‐blockers may serve as an adjunctive drug, both for stabilization of sinus rhythm in new‐onset AF patients with successful conversion, and for prevention of AF occurrence in those without new‐onset AF during hospitalization. Additionally, new‐onset AF was still an independent risk of AF at 1 year, supporting close rhythm monitoring for all patients with new‐onset AF, whether successful conversion was once achieved or not. Collectively, in patients without evidence of atrial arrhythmia before AV surgery, new‐onset AF could serve as a warning sign of long‐term AF, which required conversion, continuous medications and monitoring. The benefit of prophylactic procedures for patients with high risk of new‐onset AF merits further investigations.

### 4.1 Limitations

There are several limitations that should be recognized in the present study. First, this is a single‐center cohort study that does not include prospective enrollment or randomization. Second, the study cohort might include patients with subclinical AF that was not detected by preoperative examinations. In addition, patients were not accessible to continuous rhythm monitoring, which might have led to underestimated rates of AF after discharge.^19^

## 5 CONCLUSIONS

New‐onset AF is a common complication after AV surgery that can be effectively managed by rhythm control therapy with a > 90% successful conversion rate. However, despite those efforts, patients with new‐onset AF are predisposed to higher risks of stroke and AF at 1 year. Continuous use of beta‐blockers may be useful to reduce recurrence of AF.

## CONFLICT OF INTEREST

The authors declare no conflict of interest.

## Supporting information


**Table S1**: Baseline and perioperative characteristics of patients with and without follow‐up data.

## Data Availability

Research data are not shared.

## References

[1] January CT , Wann LS , Alpert JS , et al. 2014 AHA/ACC/HRS guideline for the management of patients with atrial fibrillation: a report of the American College of Cardiology/American Heart Association task force on practice guidelines and the Heart Rhythm Society. J Am Coll Cardiol. 2014;64:e1‐e76. 24685669 10.1016/j.jacc.2014.03.022

[2] Melby SJ , George JF , Picone DJ , et al. Time‐related parametric risk factor analysis for postoperative atrial fibrillation following heart surgery. J Thorac Cardiovasc Surg. 2015;149:886‐892. 25534301 10.1016/j.jtcvs.2014.11.032

[3] Kalra R , Patel N , Doshi R , Arora G , Arora P . Evaluation of the incidence of new‐onset atrial fibrillation after aortic valve replacement. JAMA Intern Med. 2019;179:1122‐1130. 31157821 10.1001/jamainternmed.2019.0205 PMC6547161

[4] Filardo G , Hamilton C , Hamman B , Hebeler RF Jr , Adams J , Grayburn P . New‐onset postoperative atrial fibrillation and long‐term survival after aortic valve replacement surgery. Ann Thorac Surg. 2010;90:474‐479. 20667332 10.1016/j.athoracsur.2010.02.081

[5] Mariscalco G , Engström KG . Postoperative atrial fibrillation is associated with late mortality after coronary surgery, but not after valvular surgery. Ann Thorac Surg. 2009;88:1871‐1876. 19932252 10.1016/j.athoracsur.2009.07.074

[6] Gillinov AM , Bagiella E , Moskowitz AJ , et al. Rate control versus rhythm control for atrial fibrillation after cardiac surgery. N Engl J Med. 2016;374:1911‐1921. 27043047 10.1056/NEJMoa1602002 PMC4908812

[7] David TE . Aortic valve sparing in different aortic valve and aortic root conditions. J Am Coll Cardiol. 2016;68:654‐664. 27491910 10.1016/j.jacc.2016.04.062

[8] Ma W , Zhang W , Shi W , Kong Y , Ma X . Left ventricular diastolic function after aortic valve replacement for chronic aortic regurgitation. Ann Thorac Surg. 2018;106:24‐29. 29673639 10.1016/j.athoracsur.2018.03.034

[9] Kirchhof P , Benussi S , Kotecha D , et al. 2016 ESC guidelines for the management of atrial fibrillation developed in collaboration with EACTS. Eur Heart J. 2016;37:2893‐2962. 27567408 10.1093/eurheartj/ehw210

[10] Tarantini G , Mojoli M , Urena M , Vahanian A . Atrial fibrillation in patients undergoing transcatheter aortic valve implantation: epidemiology, timing, predictors, and outcome. Eur Heart J. 2017;38:1285‐1293. 27744287 10.1093/eurheartj/ehw456

[11] Saxena A , Shi WY , Bappayya S , et al. Postoperative atrial fibrillation after isolated aortic valve replacement: a cause for concern? Ann Thorac Surg. 2013;95:133‐140. 23200233 10.1016/j.athoracsur.2012.08.077

[12] Banach M , Goch A , Misztal M , Rysz J , Jaszewski R , Goch JH . Predictors of paroxysmal atrial fibrillation in patients undergoing aortic valve replacement. J Thorac Cardiovasc Surg. 2007;134:1569‐1576. 18023685 10.1016/j.jtcvs.2007.08.032

[13] Motloch LJ , Reda S , Rottlaender D , et al. Postprocedural atrial fibrillation after transcatheter aortic valve implantation versus surgical aortic valve replacement. Ann Thorac Surg. 2012;93:124‐131. 22115334 10.1016/j.athoracsur.2011.08.078

[14] Miceli A , Murzi M , Gilmanov D , et al. Minimally invasive aortic valve replacement using right minithoracotomy is associated with better outcomes than ministernotomy. J Thorac Cardiovasc Surg. 2014;148:133‐137. 24035370 10.1016/j.jtcvs.2013.07.060

[15] Siontis GCM , Praz F , Lanz J , et al. New‐onset arrhythmias following transcatheter aortic valve implantation: a systematic review and meta‐analysis. Heart. 2018;104:1208‐1215. 29275399 10.1136/heartjnl-2017-312310

[16] Axtell AL , Moonsamy P , Melnitchouk S , et al. Preoperative predictors of new‐onset prolonged atrial fibrillation after surgical aortic valve replacement. J Thorac Cardiovasc Surg. 2020;159(4):1407‐1414. 31204133 10.1016/j.jtcvs.2019.04.077

[17] Swinkels BM , de Mol BA , Kelder JC , Vermeulen FE , Ten Berg JM . New‐onset postoperative atrial fibrillation after aortic valve replacement: effect on long‐term survival. J Thorac Cardiovasc Surg. 2017;154:492‐498. 28390762 10.1016/j.jtcvs.2017.02.052

[18] Singh BN , Singh SN , Reda DJ , et al. Amiodarone versus sotalol for atrial fibrillation. N Engl J Med. 2005;352:1861‐1872. 15872201 10.1056/NEJMoa041705

[19] Sanna T , Diener HC , Passman RS , et al. Cryptogenic stroke and underlying atrial fibrillation. N Engl J Med. 2014;370:2478‐2486. 24963567 10.1056/NEJMoa1313600

