# Supplementary material for: Rhythm outcomes after aortic valve surgery: Treatment and evolution of new‐onset atrial fibrillation
Source: Clin Cardiol. 2021 Aug 14;44(10):1432–9. doi: 10.1002/clc.23703 (PMC8495075; doi:10.1002/clc.23703)
Supplement: Supplementary file 1 — Table S1: Baseline and perioperative characteristics of patients with and without follow‐up data. [file CLC-44-1432-s001.doc]

**Supplemental Materials**

**Supplemental Tables**

Supplemental Table 1. Baseline and perioperative characteristics of patients with and without follow-up data.

| Demographics | With follow-up  (n=918) | Without follow-up  (n=60) | P value |
| --- | --- | --- | --- |
| Male sex | 626 (68.2) | 44 (73.3) | 0.41 |
| Age | 59 (48-66) | 58 (51-65) | 0.77 |
| Diabetes | 66 (7.2) | 3 (5.0) | 0.79^a^ |
| Hypertension | 395 (43.0) | 20 (33.3) | 0.14 |
| Coronary artery disease | 69 (7.5) | 6 (10.0) | 0.45^a^ |
| Chronic lung disease | 24 (2.6) | 1 (1.7) | >0.99^a^ |
| Cerebrovascular disease | 44 (4.8) | 1 (1.7) | 0.52^a^ |
| Chronic kidney disease | 16 (1.7) | 2 (3.3) | 0.30^a^ |
| Peripheral artery disease | 12 (1.3) | 4 (6.7) | 0.013^a^ |
| Connective tissue disorder | 17 (1.9) | 0 (0) | 0.62^a^ |
| Autoimmune disease | 22 (2.4) | 1 (1.7) | >0.99^a^ |
| New York Heart Association functional class III–IV | 686 (74.7) | 48 (80.0) | 0.36 |
| Heart rate (beats per minute) | 69 (63-77) | 69 (63-80) | 0.62 |
| Left atrial dimension (mm) | 40 (37-44) | 41.9±6.4 | 0.20 |
| Left ventricular ejection fraction (%) | 62 (56-66) | 61 (51-65) | 0.22 |
| Bicuspid aortic valve | 396 (43.1) | 32 (53.3) | 0.12 |
| Aortic stenosis | 165 (18.0) | 16 (26.7) | 0.093 |
| Aortic regurgitation | 510 (55.6) | 29 (48.3) | 0.28 |
| Aortic stenosis + regurgitation | 243 (26.5) | 15 (25.0) | 0.80 |
| Mitral regurgitation >mild | 17 (1.9) | 5 (8.3) | 0.009^a^ |
| Tricuspid regurgitation >mild | 13 (1.4) | 2 (3.3) | 0.23^a^ |
| Ascending aortic diameter >40 mm | 490 (53.4) | 31 (51.7) | 0.80 |
| Approaches |  |  |  |
| Sternal | 819 (89.2) | 52 (86.7) | 0.54 |
| Supra-sternal | 71 (7.7) | 8 (13.3) | 0.14^a^ |
| Right-thoracic | 28 (3.1) | 0 (0) | 0.41^a^ |
| Bioprosthesis | 344 (37.5) | 16 (26.7) | 0.093 |
| Prosthetic size | 23 (21-25) | 23 (21-25) | 0.57 |
| Surgical aortic valve repair | 62 (6.8) | 0 (0) | 0.028^a^ |
| Ascending aortic/root replacement | 330 (36.0) | 17 (28.3) | 0.23 |
| In-hospital mortality | 6 (0.7) | 0 (0) | >0.99^a^ |
| Morbidities | 49 (5.3) | 2 (3.3) | 0.76^a^ |
| Low cardiac output | 10 (1.1) | 1 (1.7) | 0.50^a^ |
| Stroke | 14 (1.5) | 0 (0) | >0.99^a^ |
| Dialysis | 5 (0.5) | 1 (1.7) | 0.32^a^ |
| Ventilator support >96 hours | 17 (1.9) | 0 (0) | 0.62^a^ |
| Reoperation for bleeding | 2 (0.2) | 0 (0) | >0.99^a^ |
| Pacemaker implantation | 16 (1.7) | 0 (0) | 0.62^a^ |
| Hypokalemia | 178 (19.4) | 7 (11.7) | 0.14 |
| Transfusion | 218 (23.8) | 15 (25.0) | 0.83 |
| Red blood cell (IU) | 0 (0-0) | 0 (0-0) | 0.89 |
| Plasma (ml) | 0 (0-0) | 0 (0-0) | 0.97 |
| Perioperative medications |  |  |  |
| Catecholamine | 822 (89.5) | 54 (90.0) | 0.91 |
| Phosphodiesterase inhibitor | 866 (94.3) | 56 (93.3) | 0.77^a^ |
| Dopamine/dobutamine | 308 (33.6) | 22 (36.7) | 0.62 |
| Levosimendan | 44 (4.8) | 5 (8.3) | 0.22^a^ |
| New-onset AF | 240 (26.1) | 16 (26.7) | 0.93 |

Continuous variables are presented as medians (interquartile ranges) or means ± standard deviations, according to the normality test. Categorical variables are presented as numbers (percentages). AF, atrial fibrillation.

^a^ Fisher’s exact test.
